# Histological Scores in Patients with Inflammatory Bowel Diseases: The State of the Art

**DOI:** 10.3390/jcm11040939

**Published:** 2022-02-11

**Authors:** Edoardo Vespa, Ferdinando D’Amico, Mauro Sollai, Mariangela Allocca, Federica Furfaro, Alessandra Zilli, Arianna Dal Buono, Roberto Gabbiadini, Silvio Danese, Gionata Fiorino

**Affiliations:** 1 Department of Gastroenterology and Hepatology, Humanitas Research Hospital, IRCCS, Rozzano, 20089 Milan, Italy; edoardo.vespa@humanitas.it (E.V.); arianna.dalbuono@humanitas.it (A.D.B.); roberto.gabbiadini@humanitas.it (R.G.); 2 Department of Biomedical Sciences, Humanitas University, Rozzano, 20089 Milan, Italy; damico_ferdinando@libero.it; 3 Gastroenterology and Endoscopy, IRCCS Ospedale San Raffaele, Vita-Salute San Raffaele University, 20132 Milan, Italy; Allocca.Mariangela@hsr.it (M.A.); zilli.alessandra@hsr.it (A.Z.); danese.silvio@hsr.it (S.D.); 4 Department of Pathology, Humanitas Research Hospital, IRCCS, Rozzano, 20089 Milan, Italy; mauro.sollai@humanitas.it; 5 IBD Center, Humanitas Research Hospital, IRCCS, Rozzano, 20089 Milan, Italy; federica.furfaro@humanitas.it

**Keywords:** histology, score, IBD, ulcerative colitis, Crohn’s disease, histological remission

## Abstract

The histological assessment has been advocated as a detailed and accurate measure of disease activity in inflammatory bowel diseases (IBD). In ulcerative colitis (UC), histological activity has been demonstrated to be associated with higher rates of relapse, prolonged corticosteroid use and long-term complications, even when endoscopic remission is achieved. Therefore, histological healing may represent a potential treatment target. Several histological scores have been developed and are available today. The Robarts histopathology index (RHI) and the Nancy index (NI) are the only two recommended by the European Crohn’s and Colitis Organization (ECCO) for use in patients with UC. Conversely, in Crohn’s disease (CD), the discontinuous nature of lesions has limited standardized histological assessment. Most of the available histological scoring systems in CD are complex and not validated. The aim of this review is to comprehensively summarize the latest evidence regarding histological scoring systems in IBD. We guide the reader through understanding the importance of an accurate microscopic evaluation using validated scoring systems, highlighting the strengths and pitfalls of each score. The priorities of future research needs are also addressed.

## 1. Introduction

The treatment of inflammatory bowel diseases (IBD) has deeply changed over recent years, due to the increasing availability of highly effective therapies, which can now target multiple pathogenic pathways [1]. At a similar pace, treatment targets in IBD have also evolved, moving from symptom control to more objective evidence of mucosal healing (MH) [2]. Robust evidence showed that endoscopic improvement was associated with improved long-term outcomes [3]. While in the last few decades, endoscopic remission has represented the most important treatment target, it was recently learned that up to one third of patients with endoscopic healing may still have microscopic disease [4,5,6,7]. Therefore, the histological assessment has been proposed as a deeper and more accurate measure of disease activity. In ulcerative colitis (UC) patients, it has been demonstrated that histological activity, even when endoscopic remission is achieved, is associated with higher rates of relapse, prolonged corticosteroid use and long-term complications, corroborating the hypothesis that histological healing may represent a potential treatment target [8,9,10,11]. In Crohn’s disease (CD), the discontinuous nature of lesions has limited the standardized histological assessment. Interest regarding CD histology been growing recently, but data are still sparse and conflicting [11]. Nevertheless, recent international consensuses recognized that achievement of histological remission is an appropriate endpoint in UC and CD clinical trials [12,13]: accordingly, the definition of MH itself may evolve, integrating endoscopic and histological improvements to better capture a deeper stage of disease control. Nevertheless, histological healing has not been formally endorsed by STRIDE-II consensus as a treatment goal in clinical practice [14]. Accumulating evidence shows that histological activity is a predictor of the disease course, especially in UC [11]. A major limitation towards a harmonized histological assessment in IBD in the past was the existence of multiple histological scoring systems. Most of them never underwent required validation, and such plurality may have contributed to confusion among pathologists, clinicians and researchers. To reliably assess microscopic activity and ensure easy adoption, an accurate, easy-to-use and reproducible score should be available, ideally [15]. Initially developed indexes are likely to be far from that definition, and only over the last few years have substantial efforts been made to standardize histological assessments of IBD, through consensus generation which promoted development and implementation of meticulously validated scoring systems [16]. The aim of this review is to comprehensively summarize the latest evidence regarding histological scoring systems in IBD. We guide the reader through understanding the importance of an accurate micro-scopic evaluation using validated scoring systems, highlighting the strengths and pitfalls of each score. The priorities of future research needs are also addressed.

## 2. Assessing Histological Disease Activity: A Guide for the Clinician

An adequate histological assessment must begin with an appropriate biopsy. In UC, the colonic mucosa is inflamed with a continuous pattern from the rectum (which is technically always involved at the diagnosis) to the cecum. In CD, the inflammation is discontinuous and may occur along the entire digestive tract from mouth to anus, although the terminal ileum is the most commonly affected. At least two biopsies from each one of the five segments of colon (right colon, transverse, descending, sigmoid and rectum) and from the terminal ileum should be obtained during the diagnostic examination of every patient with suspected IBD. To maximize diagnostic yield and accurately assess inflammation, additional biopsies should be taken from the endoscopically most affected areas—in particular, at ulcers’ edges if these are found [16,17,18]. Samples should then be sent in separate vials corresponding to different anatomical sites, as localization is important to put microscopic features in context (eosinophils, for instance, are highly represented in the left colon) and to correctly assess microscopic disease extent [16].

There are typical microscopic features of IBD which are key to making the diagnosis and defining the level of histological activity that should always be reported by pathologists to allow for a standardized histological grading of disease activity [16]. Such features have been differently combined to build up several histological scoring systems. Although showing slight differences, the main histological features of UC activity are shared with CD, so that the same histological indexes are now being applied in clinical trials, and new ones are being designed to be applied to both diseases [19,20,21]. We briefly summarize the main microscopic features of IBD and the evidence to support their inclusion in histopathologic reports.

### 2.1. Neutrophils

**Figure 1:**
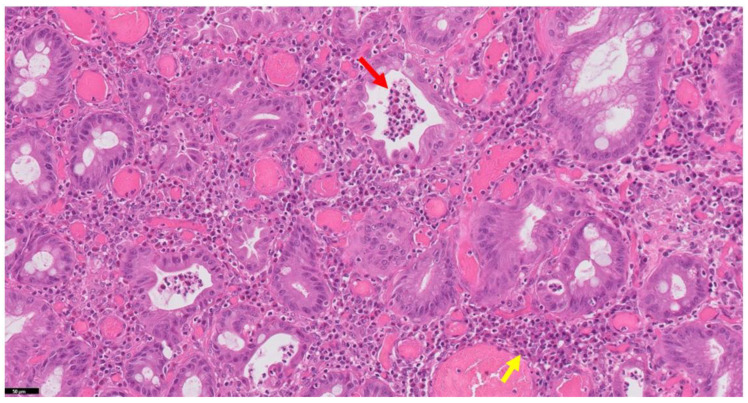
Histology of ulcerative colitis with activity features (H&E stain). Note the typical features: crypt abscesses (neutrophils contained in the crypt lumen—red arrow) and chronic inflammatory infiltrate (yellow arrow).

The landmark feature of histological activity in IBD is defined by the presence of mucosal neutrophilic inflammation within the lamina propria, the surface epithelium, in the crypt epithelium (defined as cryptitis) and the lumens of crypts (crypt abscesses) [16] (Figure 1). Neutrophilic epithelial infiltrate is never present in a normal colon and is the landmark of acute inflammation. The presence of neutrophils reflects histological activity, and there is growing consensus that, in a dichotomous and simple manner, neutrophils’ absence may correspond to histological remission [16]. There is evidence that the quickness of response to treatment in terms of neutrophilic inflammation plays a crucial role in predicting future disease course: a recent post-hoc analysis of VARSITY trial showed that elevated neutrophils in the epithelium was the only histological parameter associated with increased odds of week 52 endoscopic improvement (OR, 3.63; 95% CI, 1.45–9.08) [22].

### 2.2. Basal Plasmacytosis

Basal plasmacytosis is defined as plasma cells located between colonic crypts and the muscularis mucosae. It is a highly specific feature for the diagnosis of IBD, especially at early stages. However, it has relatively low sensitivity, as it can be focal and can be missed by a biopsy [15,16]. Of note, basal plasmacytosis may predict disease relapse in IBD. A recent meta-analysis investigated the impacts of histological factors on clinical outcomes, showing that basal plasmacytosis was individually associated with clinical relapse, even in patients with endoscopic remission (OR 1.94, 95% CI 1.10–3.46) [11].

### 2.3. Lamina Propria Chronic Inflammatory Cell Density

This feature plays a key role in the diagnosis of IBD, and to be deemed as pathological, an unequivocal increase in lymphocytes and plasma cells within the lamina propria must be present [16]. A mild increase in lamina propria chronic inflammatory cell density is usually not accompanied by basal plasmacytosis. 

### 2.4. Eosinophils

Evaluation of mucosal eosinophilic infiltrate may be challenging for pathologists, as its level varies throughout the colon in normal individuals (in which it may be present as opposed to neutrophils). It is present at higher densities in the right colon [23]. Unfortunately, a clear cut-off defining a pathological increase colonic lamina propria eosinophils is lacking [24]. Their significance as an indicator of histological activity is therefore unclear. Recently, a study by Kim and colleagues investigated the associations between isolated eosinophil density, and disease activity and treatment response: interestingly, 65 IBD patients on vedolizumab which were not responsive to treatment had a significantly higher baseline mean eosinophil count than those who responded (340 ± 156 vs. 236 ± 124; *p* = 0.004) [25].

### 2.5. Erosions and Ulcers

Surface epithelial damage is unanimously considered the most severe sign of acute inflammation, and if present, usually the highest grade of activity of any scoring system is given [15,16]. Mucosal breaks are defined as the loss of normal epithelium, which is replaced with granulation tissue or by clearly evident fibrinous exudate. The distinction between ulcers and erosions is that the former typically extends deeper than the muscularis mucosae, whereas the latter does not; but for the pathologist it can be hard to tell the difference in practice.

### 2.6. Crypt Architectural Distortion

**Figure 2:**
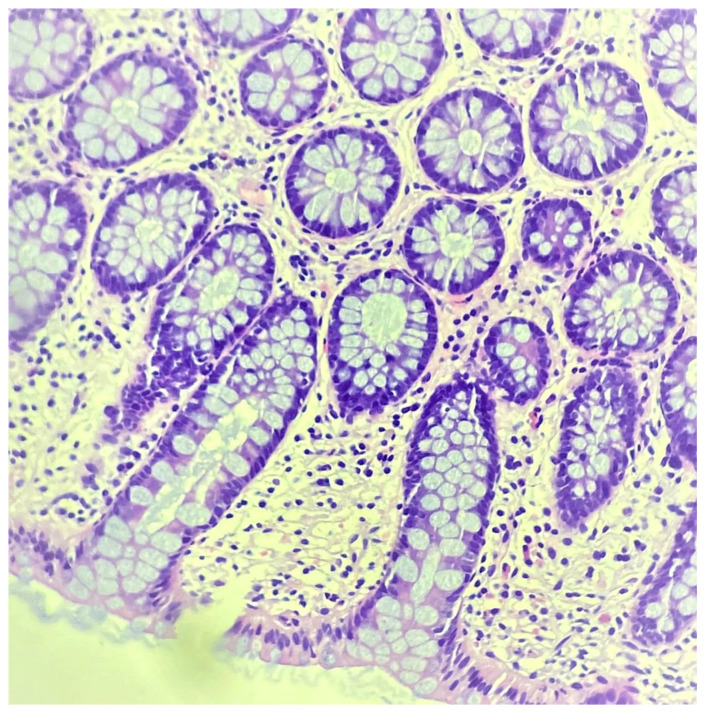
Histology of ulcerative colitis with remission features. Only mild crypt architectural distortions are evident.

Features of crypt distortion include branching, loss of parallelism, irregularity, tortuosity, dilatation and variations in shape and size [16]. It is well known that rare architecturally distorted crypts can be identified in a normal colon and can also be found in histologically quiescent IBD (Figure 2). Furthermore, it is common histopathological knowledge that crypts in the rectum often do not reach the muscularis mucosae. Although architectural abnormalities alone may not contradict histological remission (as long as no inflammatory features are present), in a recent meta-analysis, crypt distortion was individually associated with higher rates of clinical relapse in patients who achieved endoscopic remission (OR 1.94, 95% CI 1.10–3.46) [11].

### 2.7. Crypt Atrophy

A typical morphological feature of atrophy is the shortening of the crypt, making larger gaps between the base and muscularis mucosae [16]. The presence of a local excess of neutrophils in a part of a crypt proves higher severity. Unequivocal crypt destruction requires loss of continuity between epithelial cells within a crypt. 

### 2.8. Mucin Depletion

It is defined as significant reduction in number of goblet cells [16]. One study showed that in patients with endoscopic remission (Mayo 0), the presence of mucin depletion was the only factor significantly and independently associated with the risk of relapse (hazard ratio, 2.18, 95% CI 1.16–5.82) [26].

## 3. Histological Scores in IBD

Truelove in 1955 first described histological changes of colonic and rectal mucosa after hydrocortisone treatment in UC [27]. Since then, more than 30 scoring systems have been developed to assess histological activity in both UC and CD, which have been extensively and systematically reviewed [28,29]. Our review focuses on scoring systems which underwent formal content validation, because of their unequivocal methodological superiority. We acknowledge that the use of highly heterogeneous, non-validated scoring systems may have represented a barrier to the development of a systematic microscopic evaluation in IBD. According to recent guidelines, only validated scoring systems should be adopted both in clinical trials and in practice, with the aim of providing researchers and clinicians with comparable results and to promote harmonization of data interpretations [16]. In line with recent evidence showing that UC and CD responses to treatment can be interchangeably measured, and recent guidelines supporting the adoption of same scoring systems for CD and UC, we do not discuss scoring systems for UC and CD separately [12,13,30]. Homogenization of histological interpretations in IBD also seems a suitable research priority, and indeed, the recently developed scoring systems are designed to be applicable to both diseases.

### 3.1. Geboes Score

The Geboes score (GS) was developed by Geboes et al. in 2000, using 99 biopsies from UC patients [31]. Despite being never formally validated, it has been used in clinical trials, and it continued to represent the histological outcome measure in recent randomized trials of biologics and small molecules [32,33]. On the other hand, there have been some barriers to the GS’s adoption in practical use, mainly due to its complexity. It evaluates seven landmark histological features of inflammatory bowel disease: the architecture of the crypts (GS 0), lamina propria chronic inflammatory infiltrate (GS 1), lamina propria eosinophils (GS 2A), lamina propria neutrophils (GS 2B), intraepithelial neutrophils (GS 3), crypt destruction (GS 4) and surface epithelial injury (GS 5), each with its own levels of severity. Each of these seven subscoring systems is evaluated separately using a 0–3 scale (i.e., GS 2B.2) except for the surface epithelial injury, which is on a 0–4 scale: higher numerical values indicate more severe inflammation (Table 1). The worst instance among available biopsies is taken, rather than an average of all specimens, helping to represent the worst activity features present in the mucosa and capturing microscopic heterogeneity. Regarding the key variables, architectural abnormalities can be found in up to 20% of healthy people according to the GS. The increase in lamina propria chronic inflammation, with focal (generally <50% of the biopsy) and diffuse (≥50%) basal plasmacytosis, configures different grades. While eosinophilic density is one of the items in the GS, it has been removed as a single scoring variable in indexes that were developed later (Robarts and Nancy index), considering its unclear status as a significant histological feature in IBD. The GS can be either used simply by assigning a score from 0 to 6 [34] based on the highest GS subscore seen in the biopsy, or in a continuous manner by summing the scores from all GS subscores to generate a score (defined as continuous GS) between 0 and 22 [35]. In 2016, a simplified GS (SGS) version was proposed, which was made to reduce its practical complexity [36]: the number of grades was reduced to six, and the degrees of activity were also reduced (0–2 for each domain, except surface damage 0–3). SGS merges grade 0 and 1 of GS, so that biopsies showing no active inflammation but residual signs of activity now fall within grade 0. Basal plasmocytosis was added as a scoring variable (grade 1) because of the evidence supporting its role as an independent predictor of disease course. Histological remission is defined as continuous GS ≤ 6 or GS ≤ 2.0 (absence of epithelial neutrophils); histological response is defined as continuous GS ≤ 12 or GS < 3.0 [16]; no validated definitions of remission and response have been provided for using the SGS so far.

### 3.2. Robarts Histopatological Index

The Robarts histopathological index (RHI) was developed in 2017, through a multiple linear regression model process followed by a bootstrap procedure [37]. It is mainly derived from the GS. It includes the four original items that showed high inter-rater and intra-rater reliability, and it strongly correlates with histological disease activity as measured on a visual analog scale. The included items are 1: lamina propria chronic inflammation; 2: lamina propria neutrophils; 3: epithelial neutrophils; 4: surface epithelial injury. Each item receives a grade of 1–4; the total score ranges from 0 (no disease activity) to 33 (most severe disease activity). There are different weights for each feature, with the lowest being for chronic inflammation and the highest being for erosion/ulceration (Table 1). In UC, histological remission is defined as RHI ≤ 3 (subscores of lamina propria neutrophils and neutrophils must be equal to 0, with no ulcers or erosions); histological response is defined as RHI ≤ 9 (subscores of lamina propria neutrophils and neutrophils must be equal to 0, with no ulcers or erosions) [16]. The RHI was recently shown to strongly correlate with continuous GS in UC (coefficient 0.806 [*p* < 0.001]), as in terms of histological remission, positive predictive values (PPVs) were satisfactory (99% and 95% for RHI > 6 and RHI > 3 or subscores > 0 for neutrophils in lamina propria and intraepithelial, respectively), meaning that that 99% and 95% of all patients classified as having RHI > 6 and an absence of histological remission according to the RHI, respectively, had histological activity according to the GS. Moreover, strong accuracy was found in the definition of a histological response: 95% and 88% of all patients classified as having and not having a histological response according to the RHI also fell into the same categories according to the GS [38]. As regards CD, the RHI has recently been used as a histological endpoint in a phase 2 trial with ozanimod, where a significant reduction was obtained after treatment, and will be a secondary endpoint in the ongoing phase 3 trial [17,NCT03464097].

### 3.3. Nancy Index

The Nancy index (NI) was developed in 2017 using 200 biopsies, to provide a scale that was developed by studying eight features and including only domains that correlated with a Global Visual Evaluation of histopathological severity. During its development it was shown to strongly correlate with GS, albeit it is simpler and has fewer variables [39]. The NI requires a stepwise evaluation of three features, lamina propria chronic inflammation (defined as lymphocytes, plasma cells and eosinophils), neutrophilic inflammation and surface damage (erosion/ulcers), to build up a continuous 0–4 score (Table 2). In contrast to the RHI, the worst feature present among biopsies determines the final score. The presence of erosions or ulcerations defines an NI of 4. If those are not found, the intensity of neutrophilic infiltrate within the mucosa is assessed: if a few neutrophils are present in lamina propria or in the epithelium, then the NI is 2. More intense inflammation with clusters of neutrophils defines an NI of 3. If no neutrophils are present, then the NI is 0 or 1: grade 1 requires the presence of a moderate increase in lamina propria lymphoplasmacytic inflammation or eosinophils. If no, or only a mild, increase in lymphocytes, plasma cells and eosinophils is observed, then the score is 0. Histological remission is defined as NI = 0 and histological response is defined as NI ≤ 1, when there are no neutrophils in the epithelium, nor erosions or ulcers. The undoubted strengths of NI, other than being fully validated, are several: Jairath et al. showed that it highly correlates with the RHI and the response to change after treatment [33]. A study by Magro et al. has demonstrated that it correlates strongly with continuous GS and with fecal calprotectin levels, a useful non-invasive biomarker [40]. Last but not least, NI is conceptaully simple and easy to apply: accordingly, the ECCO recommends its use in clinical practice [16].

### 3.4. IBD-DCA Score

The need for a score which is simple, reproducible and applicable to both UC and CD led to the development in 2021 of the Inflammatory Bowel Disease—Distribution, Chronicity, Activity (IBD-DCA) score [21]. Differently to NI and RHI, which mostly include features of inflammation, chronic and architectural features have been included in the IBD-DCA score. Crypt architectural distortion has indeed been suggested to be a crucial feature, as it may help to distinguish between quiescent UC (which has architectural distortions) and true histological normalization (normal colon). Moreover, an association between crypt architectural changes and increased risk of clinical relapse has been found [11]. Three key features are evaluated in this score: (D) the distribution of either chronic or active histopathological modifications, regardless of their nature (architectural, epithelial or inflammatory); (C) chronicity parameters (architectural distortion or chronic inflammation); (A) activity parameters (neutrophils) (Table 3). Grading is given in the following stepwise fashion: whereas D0 corresponds to normality features in scanning magnification (2.5–4×), D1 and D2 correspond to any active or chronic histological changes from normal, irrespective of their nature. In the second step, chronic injury is assessed: C0 is defined as normality; C1 refers to isolated crypt distortion or mild lymphoplasmacytosis; C2 refers a marked increase in lymphoplasmacytic lamina propria infiltration, regardless of crypt abnormalities. In the third step, active inflammation is evaluated: A0 corresponds to the absence of any feature of active inflammatory damage; A1 corresponds to mild inflammatory activity with two or more neutrophilic granulocytes in one high-power field (HPF) in the lamina propria or any neutrophils in the epithelium; A2 includes crypt abscesses or features of epithelial surface damage (erosions and ulcerations). Inter-rater agreement was moderate–good for a UC cohort and at best moderate for a CD cohort. Intra-rater agreement was good to excellent in both cohorts. The correlation with the SGS was moderate to strong. Limitations of the IBD-DCA score are that it still needs prospective validation and that it lacks a clear definition of histological response/remission: in detail, whereas the authors suggest that C1 grade may entirely correspond to remission (NI 0, SGS 0.1–1.1), the significance of C2 grade is less clear (marked lymphoplasmacytosis or basal plasmocytosis) regarding remission status (it should correspond to NI 1–2, SGS 1.2). However, the addition to the pathologist’s armamentarium of a rational and simple score such as IBD-DCA, validated for both UC and CD, has to be welcome, and future investigation is warranted.

In conclusion, as validated scoring systems have been introduced, implementation of a standardized histological assessment in IBD is a priority and should not be further delayed. Among those available, NI provides clarity, replicability and ease of use, so that its use in clinical practice is now recommended by the ECCO [16].

## 4. Histologic Healing in IBD: Towards Disease Clearance?

The use of reliable and practical scoring systems to measure histological activity is now a priority, as a growing body of evidence demonstrates that persistent microscopic inflammation may be associated with adverse long-term outcomes. There is enough rationale to postulate that, if disease activity is microscopically controlled, a substantial alteration to its future course can follow. Whereas the evidence in UC is becoming robust, there is a need for further research in CD: the literature is summarized below. 

In a landmark study, Bryant et al. included 91 UC patients, stratified by histological and endoscopic activity, and investigated the correlations of UC with corticosteroid use, hospitalization and colectomy over a long-term follow-up period (over 5 years): the absence of histological activity was associated with corticosteroid use (hazard ratio = 0.42; 95% confidence interval (CI): 0.2–0.9) and hospitalization (hazard ratio = 0.21; 95% CI: 0.1–0.7) [41]. Another study by Lobaton et al. showed that in 96 patients with active histology assessed by GS, rates of clinical relapse at 1 year were higher, with an OR of 4.29 (95% CI 1.55–11.87) for GS ≥ 2B.1 and 4.31 (95% CI 1.52–12.21) for GS ≥ 3.1 [42]. Magro et al., in a larger sample of 399 asymptomatic patients, showed that higher grades of histological activity as measured by GS are associated with incremental risks of UC progression over 36 months (a composite endpoint of surgery, pharmacologic escalation, corticosteroid use, hospitalization): patients with GS > 2B.0, GS > 3.0 or GS > 4.0 were more likely to show UC progression, more quickly, than patients with GS ≤ 2B.0, GS ≤ 3.0 or GS ≤ 4.0 (*p* < 0.001). Disease progression also occurred earlier in patients with GS > 2B.0, GS > 3.0 or GS > 4.0 compared with patients with GS ≤ 2B.0 (HR, 2.021; 95% CI, 1.158–3.526), GS ≤ 3.0 (HR, 2.007; 95% CI, 1.139–3.534) or GS ≤ 4.0 (HR, 2.349; 95% CI, 1.269–4.349) [43]. D’Amico et al. demonstrated similar results using NI: of the 186 patents included, a significantly higher percentage of patients with baseline activity (NI ≥ 1) underwent colectomies over the course of 1 year as compared with those in remission (14.0% vs. 0.0%, *p* = 0.01). The hospitalization rate was also found to be higher in patients with histological activity at baseline than those in remission (36.0% vs. 7.1%, *p* = 0.001) [44]. These results have been recently confirmed in a meta-analysis by Gupta et al., including 2677 UC patients in endoscopic remission, which demonstrated a 58% reduction in relapses in patients with histological remission (both assessed by GS or NI) as compared to those with persistent inflammation, with a comparable measure among those with Mayo 0 and Mayo 0/1 endoscopic features [11].

We discussed earlier that the association seems less clear in CD than in UC, although this could just be due to the paucity of data collected so far. Some studies have addressed the issue, but with conflicting results: Christensen et al. showed that ileal CD patients in clinical remission, with histological healing but not endoscopic healing, had better clinical outcomes, including less clinical relapse (HR, 2.05; 95% CI, 1.07–3.94), medication escalation (HR, 2.17; 95% CI, 1.2–3.96) and corticosteroid use (HR, 2.44; 95% CI, 1.17–5.09) [45]. According to Yoon et al., patients with clinical and endoscopic remission who were also in histological remission had a 43% lower risk of treatment failure (1-year cumulative risk: 12.9% vs. 18.2%; adjusted hazard ratio, 0.57 (95% confidence interval, 0.35–0.94)) as compared to those with persistent histological activity [46]. On the other hand, Hu et al. demonstrated that histological healing in the ileum or colon was not associated with reduced clinical relapse rates in patients with both clinical and endoscopic remission [47]. To summarize, whereas two studies showed a stronger association between histological healing in the ileum and subsequent relapse, as compared to endoscopic healing alone, another failed to demonstrate that, if endoscopic remission is achieved, histological activity still plays a prognostic role.

Data also suggest that finer microscopic control of inflammation can prevent epithelial dysplastic modifications and carcinogenesis in both UC and CD [48,49]. Recently, Kirchgesner et al. performed a case–control study including 45 patients with neoplasia (dysplasia/adenocarcinoma, 20 CD and 25 UC) and 353 controls: histological activity as assessed by NI was associated with an increased risk of colorectal neoplasia (per 1-unit increase, OR, 1.69; 95% CI, 1.29–2.21) [50].

**Figure 3:**
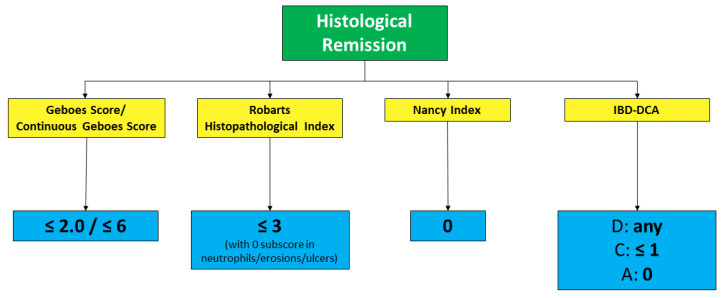
Histological remission definition across validated scoring systems for UC (the two values for Geboes Score differ depending on whether original or continuous grading is used).

**Figure 4:**
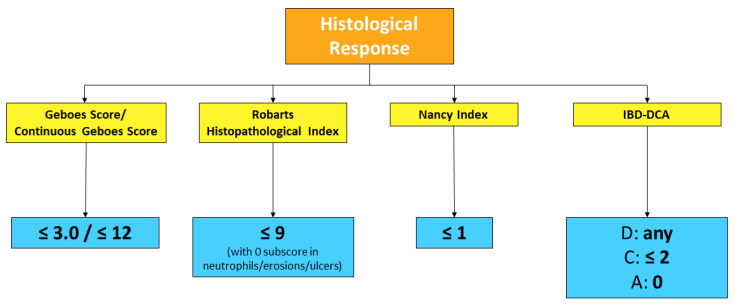
Histological response definitions across validated scoring systems for UC (the two values for Geboes Score differ depending on whether original or continuous grading is used).

In conclusion, in UC, the groundwork seems to have been done for accepting histological remission as the new therapeutic target. Going beyond endoscopic healing, and even better, integrating it, may lead to finer disease control, potentially changing one’s subsequent disease course, to eventually reach “disease clearance” [51]. Our group recently showed, in a retrospective multicenter analysis including 302 patients, that 51 patients (16.9%) who achieved both endoscopic and histological remission experienced significantly lower rates of hospitalization (7.8% vs. 25.9%, *p* = 0.008) and surgery (0.0% vs. 8.8%, *p* = 0.05) compared with patients with endoscopic and/or histological disease activity. Kaplan Meier curves confirmed that patients with disease clearance at baseline had lower risks of surgery (*p* = 0.04) and hospitalization (hazard ratio (HR) = 0.49, 95% confidence interval (CI) 0.08–2.29, *p* = 0.009) [52]. As in clinical practice, this target may still seem ambitious, we suggest that histological activity should be taken into account when decision-making is doubtful (for instance, whether to escalate treatment or not). Currently accepted definitions of histological remission and response in UC, using validated scoring systems, are outlined in Figure 3 and Figure 4. Further data need to be collected in CD before drawing firm conclusions. 

## 5. Conclusions

The development of different scoring systems may have so far represented a barrier to the accumulation of homogeneous histological information in IBD. This may have been responsible for the delay in the formulation of standardized definitions of histological activity, response, and remission, which have only recently been formulated for UC, and that are still lacking for CD. Moreover, as the prognostic significance of histology for IBD is now undeniable, clinicians must be aware that histological remission may soon become a realistic treatment target. Thus, further data need to be collected, and a systematic implementation of validated scoring systems applicable both to UC and CD in clinical trials and practice should be pursued. Though many steps have been made in the right direction, there is still a need to identify clear histological endpoints that will predict long-term improvement and suggest disease abatement. Moreover, commonly accepted and validated histological scoring systems are needed to stratify patients who might benefit from therapy escalation or de-escalation, predicting individual risk of neoplasia development and selecting the optimal endoscopic surveillance timing.

## Figures and Tables

**Table 1:** Geboes score (GS) and the derived Robarts histopathological index (RHI).

GS	Morphology	RHI
Grade 0: Architectural changes	0.0 No abnormality	0
	0.1 Mild abnormality	0
	0.2 Mild/moderate diffuse or multifocal abnormalities	0
	0.3 Severe diffuse or multifocal abnormalities	0
Grade 1: Chronic inflammatory infiltrate	1.0 No increase	0
	1.1 Mild but unequivocal increase	1
	1.2 Moderate increase	2
	1.3 Marked increase	3
Grade 2A: Eosinophils in lamina propria	2A.0 No increase	0
	2A.1 Mild but unequivocal increase	0
	2A.2 Moderate increase	0
	2A.3 Marked increase	0
Grade 2B: Neutrophils in lamina propria	2B.0 No increase	0
	2B.1 Mild but unequivocal increase	2
	2B.2 Moderate increase	4
	2B.3 Marked increase	6
Grade 3: Neutrophils in epithelium	3.0 None	0
	3.1 <5% crypts involved	3
	3.2 <50% crypts involved	6
	3.3 >50% crypts involved	9
Grade 4: Crypt destruction	4.0 None	0
	4.1 Probable–Local excess of neutrophils in part of the crypts	0
	4.2 Probable–Marked attenuation	0
	4.3 Unequivocal crypt destruction	0
Grade 5: Erosions and ulcerations	5.0 No erosion, ulceration or granulation tissue	0
	5.1 Recovering epithelium + adjacent inflammation	5
	5.2 Probable erosion—focally stripped	5
	5.3 Unequivocal erosion	10
	5.4 Ulcer or granulation tissue	15

GS: histological remission ≤ 2.0, histological response ≤ 3.0. RHI: histological remission ≤ 3, histological response ≤ 9.

**Table 2:** Nancy index.

Grade	Morphology
0	No or only mild increase in chronic inflammatory cells
1	Moderate or severe increase in chronic inflammatory
	cells (lymphocytes, plasma cells, and eosinophils)
	defined as presence of an increase in chronic
	inflammatory cells that is easily apparent
2	Mild increase in neutrophils defined as few or rare
	neutrophils in lamina propria or in the epithelium
	that are difficult to see
3	Moderate or severe increase neutrophils defined as
	presence of multiple clusters of neutrophils in lamina
	propria and/or in epithelium that are easily apparent
4	Ulcers or erosions defined as loss of colonic crypts
	replaced with “immature” granulation tissue (disorganized blood vessels with extravasated neutrophils) or the presence of fibrinopurulent exudate

Histological remission = 0; histological response ≤ 1.

**Table 3:** IBD-DCA score.

**Parameter**	**Morphology**
Distribution [D]	0 Normal
	1 <50% of tissue affected per same biopsy site
	2 >50% of tissue affected per same biopsy
Chronic features [C]	0 Normal
	1 Crypt distortion and/or mild lymphoplasmacytosis
	2 Marked lymphoplasmacytosis and/or basal plasmacytosis
Activity features [A]	0 Normal
	1 Two or more neutrophils in lamina propria in one high-power field [HPF] and/or intraepithelial neutrophils [any number] 2 Crypt abscesses, erosions, ulcers

## Data Availability

Not applicable.
